# Very preterm infants engage in an intervention to train their control of attention: results from the feasibility study of the Attention Control Training (ACT) randomised trial

**DOI:** 10.1186/s40814-021-00809-z

**Published:** 2021-03-12

**Authors:** Oliver Perra, Sam Wass, Alison McNulty, David Sweet, Kostas A. Papageorgiou, Matthew Johnston, Delfina Bilello, Fiona Alderdice

**Affiliations:** 1 School of Nursing and Midwifery, Queen’s University Belfast, Medical Biology Building, 97 Lisburn Road, Belfast, BT9 7BL UK; 2 Centre for Evidence and Social Innovation, Queen’s University Belfast, Medical Biology Centre, 97 Lisburn Road, Belfast, BT9 7BL Northern Ireland, UK; 3 School of Psychology, University of East London, London, UK; 4 TinyLife, The Premature Baby Charity for Northern Ireland, Belfast, Northern Ireland, UK; 5 Health and Social Care Belfast Trust, Belfast, Northern Ireland, UK; 6 School of Psychology, Queen’s University Belfast, Belfast, UK; 7 Nuffield Department of Population Health, University of Oxford, Oxford, UK

**Keywords:** Infant, Premature, Feasibility study, Attention, Computerised cognitive training, Eye-tracking methodology

## Abstract

**Background:**

Very premature birth (gestational age between 28 and 31 + 6 weeks) is associated with increased risk of cognitive delay and attention deficit disorder, which have been linked to anomalies in the development of executive functions (EFs) and their precursors. In particular, very preterm (VP) infants display anomalies in controlling attention and gathering task-relevant information. Early interventions that support attention control may be pivotal in providing a secure base for VP children’s later attainments. The Attention Control Training (ACT) is a cognitive training intervention that targets infants’ abilities to select visual information according to varying task demands but had not been tested in VP infants. We conducted a feasibility study to test the processes we intend to use in a trial delivering the ACT to VP infants.

**Methods and design:**

We tested recruitment and retention of VP infants and their families in a randomised trial, as well as acceptability and completion of baseline and outcome measures. To evaluate these aims, we used descriptive quantitative statistics and qualitative methods to analyse feedback from infants’ caregivers. We also investigated the quality of eye-tracking data collected and indicators of infants’ engagement in the training, using descriptive statistics.

**Results:**

Twelve VP infants were recruited, and 10 (83%) completed the study. Participants’ parents had high education attainment. The rate of completion of baseline and outcome measures was optimal. VP infants demonstrated engagement in the training, completing on average 84 min of training over three visits, and displaying improved performance during this training. Eye-tracking data quality was moderate, but this did not interfere with infants’ engagement in the training.

**Discussion:**

The results suggest the ACT can be delivered to VP infants. However, challenges remain in recruitment of numerous and diverse samples. We discuss strategies to overcome these challenges informed by results of this study.

**Trial registration:**

Registered Registration ID: NCT03896490. Retrospectively registered at Clinical Trials Protocol Registration and Results System (clinicaltrials.gov).

**Supplementary Information:**

The online version contains supplementary material available at 10.1186/s40814-021-00809-z.

## Key messages regarding feasibility

- The feasibility of delivering a cognitive training programme, the Attention Control Training (ACT), to infants born very preterm (28 to < 32 gestational weeks) may be hindered by challenges inherent in recruiting this hard-to-reach population, as well as challenges related to the vulnerability of this population to health, cognitive and behavioural difficulties.
- The findings indicate that recruitment of very preterm infants and their families in the ACT programme presents key challenges, which also involve recruitment of families from diverse backgrounds. Nonetheless, retention was adequate and procedures such as randomisation and assessment batteries were acceptable. Notably, the findings indicate that very preterm infants engaged adequately with the programme.
- These findings indicate that recruitment strategies could benefit from involvement of gatekeepers, and strategies to build trusting relationships between researchers and participants. Infants’ engagement with the training programme suggests it may be possible to increase the number of training visits.

## Background

Advances in obstetric care since the 1990s, particularly the use of antenatal corticosteroids and surfactants, have contributed to the increasing survival and improved health of children born premature [9, 10]. However, even with these developments, premature children are still at increased risk for intellectual deficits [17, 49, 71] and poorer school attainment [2]. These risks may curtail the longer-term wellbeing of the increasing number of premature children who survive.

Infants born between 28 and less than 32 weeks gestation age and known as very premature (VP) are at increased risk of significant intellectual deficits [8, 17, 32, 34, 39, 46, 60], learning difficulties [1, 38], attention problems [1, 27, 66], problem behaviours [5, 14, 22, 42], and developmental disorders such as attention deficit with hyperactivity disorder (ADHD) [15, 16, 29, 30, 47].

Moiseev and colleagues suggested that increased risks for delays in behaviour regulation, cognitive and intellectual abilities experienced by VP infants may be linked to disruption of pre- and frontal-cortex maturation caused by very premature birth [43]. However, longitudinal studies suggest that maturation issues alone cannot explain the long-term delays of children born VP. In fact, increasing evidence suggests that VP delays may result from cumulative deficits that start accruing from an early age. In particular, VP infants display marked problems in directing, allocating and inhibiting attention in order to complete experimental tasks [25, 66], and findings published by Rose and colleagues suggest that in the first year of life VP infants display inefficient and more immature patterns of attention in tasks that require *effortful* processing of stimuli [56–58]. Collectively, these findings suggest that VP infants display difficulties in attention control—that is, the ability to select actively what to pay attention to and what to ignore. Attention control is considered a key precursor of executive functions (EFs) [3, 33], which are top-down cognitive processes that regulate other cognitive process (e.g. selection of task-relevant information) in order to fulfil a goal [23, 24]. EFs play a key role in cognitive development, as well as the development of behaviour regulation. Thus, early appearing anomalies in attention control may initiate a cascade of effects, such as poorer EFs, ultimately leading to cognitive deficits [36, 37, 41, 54, 55].

The developmental perspective outlined highlights the importance of early interventions that target foundational attention control skills. Cognitive training programmes that allow infants and young children to practise key components of their emerging cognitive abilities can succeed by capitalising on the plasticity of neural networks controlling key cognitive skills, and the fact that anomalies or deficits are not yet entrenched [13, 75]. A systematic review and meta-analysis of cognitive training programmes supports the hypothesis of an augmented effect at younger ages of delivery [78].

A promising cognitive training tailored for infants is the Attention Control Training (ACT) [6, 74]. This training programme involves showing on a screen cartoon-like characters that move and change in response to infants’ direction of gaze. This is possible thanks to an eye-tracking device that feeds information in real-time to a computer, which in turn generates adaptive visual displays. The computer programme capitalises on infants’ interest for visual and auditory stimuli and engages infants in game-like situations, whereby displaying a specific behaviour triggers a ‘reward’ stimulus, e.g. an animated character appearing and making a funny noise. Commonly, the games involve rewarding infants for learning to control their gaze away from a more salient (‘automatically’ attention-eliciting) area of the screen, in favour of using their endogenous (voluntary) attention control to orient towards a less salient area. In a different task set, infants have to scan a series of colourful targets on the screen in order to detect a pre-specified target object, which triggers the reward animation. In this way, the programme can challenge infants in using attention in flexible and increasingly sophisticated ways to obtain the reward animation. Previous studies involving typically developing infants suggest effectiveness and generalisability of the ACT programme, at least in the short term [6, 28, 74, 76].

These findings suggest that VP infants may take advantage of this intervention, which could support the emerging development of attention control abilities that are particularly challenged in this population and are pivotal for the development of Executive Functions. Evidence shows that attention control training programmes can effectively improve abilities in this domain, producing transfer of effects that is more pronounced when these abilities are targeted at a younger age [75]. Furthermore, a recent meta-analysis indicates that effects of computerised training programmes may be larger in developmentally at-risk groups, who may be more susceptible to training that targets key abilities [61]. Since VP infants may be generally more susceptible to environmental influences [62], it is possible they may benefit particularly from early interventions that address abilities at the basis of later attainment, such as attention control.

However, the implementation of the ACT with VP infants necessitates testing some of the underlying processes to be used in a randomised trial. VP infants represent a small population: in high-income countries, VP births account for approximately 1% of all births [21], and 0.8% of all births in England and Wales were VP in 2018 ([48], 2019). Furthermore, VP infants are at risk of health problems and complications [7], and VP birth is also associated with increased likelihood of parents’ mental health issues [20, 69, 70]. Furthermore, VP births are more prevalent among households in deprived areas [11, 63]. These characteristics make families of VP infants a hard-to-reach population. We thus aimed to study and resolve challenges in recruiting and retaining a sample of VP infants. Furthermore, VP infants may be more likely to display a difficult temperament [40] and poorer ability to sustain attention [53], which may hinder their ability to engage with the ACT tasks and other types of lab-based assessments.

Differences in temperament, irritability and fidgetiness may also affect the quality of eye-tracking data collected from this population: abrupt head movements and changes in posture can hinder the correct identification of the pupil and the head position, on which eye-tracking data rely. Although the ACT training is not affected by quality of the eye-tracking data [6], lower quality of eye-tracking data can affect results of eye-tracking outcome tasks, introducing biases that can inflate differences in performance [77]. Since VP infants may be more fidgety and restless than typically developing infants, comparisons in tasks that rely on eye-tracking data must ensure that the quality of data collected is comparable across groups.

### Aims of the study

There are different intervention programmes that aim to improve cognitive abilities of preterm infants [64], but few target exclusively the infants and their abilities. Furthermore, to the best of our knowledge, computerised training programmes have been tested with ex-preterm children [4, 72], but not with ex-preterm infants and toddlers. Finally, few studies have used eye-tracking methods to examine preterm infants’ attention abilities [25, 35, 59, 67]. Our overarching aim was thus to test the feasibility of delivering the computerised ACT to VP infants. In particular, our objectives were to test (a) the recruitment process; (b) retention in the programme; (c) number and duration of VP infants’ completed training/control sessions and VP infants’ engagement in the programme; (d) acceptability and completion of baseline and outcome assessments; (e) quality of eye-tracking data collected and (f) feedback from parents regarding obstacles and facilitators to study participation.

The a priori criteria set for determining success of feasibility (see [50]) were (1) recruitment of 20 VP infants in the study; (b) retention of 80% of the sample, defined as in point *d* below; (c) infants retained in the study to complete at least two training or control weekly visits, defined as attending at least two tasks for 240 s and (d) infants retained in the study to complete at least 50% of the pre- and post-test tasks.

To control for preterm infants’ maturation, we considered VP infants’ corrected age, i.e. their age adjusted for total age since conception. Using corrected age ensures that premature infants are compared to term infants that are at similar developmental and maturational stages.

## Methods

### Trial design

The study followed the protocol described in a previous publication [50] and involved a feasibility randomised trial whereby eligible infants were allocated with 1:1 ratio to either the Attention Control Training (ACT) or a control procedure. Families and infants in both arms of the study were invited to take part in five sessions for five consecutive weeks, matching the protocol used in previous studies [6, 73]. Researchers attempted to schedule the first session around the time the infant was 12 months (corrected age). The first session involved a baseline assessment of attention and cognitive skills carried out by a blinded assessor (see ‘Blinding’). If the infant was in a calm and alert state at the end of the baseline assessment, the experimenters attempted to deliver the training or control programme within this first visit. If the infant was not calm or alert, or the parents did not agree to stay for the training/control procedure, the first training or control visit was scheduled for the following weekly session.

In weeks 2, 3 and 4 of the study, the infant participated in the training or control procedure, according to their random group allocation. In week 5, infants completed the same battery of tasks delivered during the baseline assessment. The baseline and post-test assessments were delivered by a post-graduate student, dubbed in the reminder of this manuscript as the *assessor*, who was blind to infants’ group allocation. Another experimenter (from now on the *experimenter*) was responsible for delivering the training or control procedure and was instructed not to divulge this information to the assessor (see ‘Randomisation’ for more details).

The study protocol initially stipulated to run all the study sessions in a dedicated room in the premises of the local collaborating charity. However, after initial feedback from parents enrolled in the study and discussion within the study steering group, we changed the protocol to allow parents to opt to conduct the training or control sessions in their own house. The goal was to facilitate participation of parents who might have had difficulties travelling to attend the weekly sessions with their child. To ensure that training or control sessions were comparable when delivered in different settings, the same set-up was used (*see* ‘Interventions’). We devised a questionnaire to collect feedback concerning parents’ experience of the study. Following suggestions by the study steering group, parents were also asked to take part in a short telephone interview to provide more detailed feedback. Consent to take part in the study did not imply consent for this interview (see ‘Ethical approval’).

In order to incentivise participation, we offered families compensation for travel expenses incurred in attending the study sessions (at 0.40 GBP per mile). Furthermore, families of infants that completed the study were offered gift vouchers for a value of 60 GBP. These incentives had been agreed in consultation with the charity to compensate for potential child-caring costs.

A steering group that included practitioners working in neonatal services and a parent representative, as well as the study group, met approximately every 2 months to monitor progress of the study. These meetings commenced before recruitment started and ended when the study was concluded and a report for the funders produced.

### Participants

Eligibility criteria were infants born very preterm (gestational age 28 to less than 32 weeks), residing in Northern Ireland, age 12 months (± 1 month) at the time of first scheduled appointment and corrected for prematurity. Exclusion criteria were infants with significant visual and/or hearing difficulties, congenital anomalies that impacted on cognitive and sensory-motor development, or participation in other trials that could affect infants’ attention and cognitive skills. These criteria excluded, for instance, children with Down syndrome or suspected cerebral palsy: the rationale was to recruit a group of VP infants with relatively homogeneous sensory-motor and cognitive skills.

Eligible participants were identified by two categories of gatekeepers: (a) collaborating neonatology practitioners in hospitals within the Belfast, South Eastern, and Northern Trust in Northern Ireland; (b) a local charity for families of premature children. We organised preliminary individual meetings between the principal investigator (OP) and practitioners to illustrate the rationale and procedures of the study and discuss ways to present the study to parents of eligible infants. Similar group meetings with charity workers were also carried out, followed by discussions. The gatekeepers ensured that parents or caregivers of eligible infants received information about the study. Parents or caregivers interested in the study had to contact the research team in order to receive more details and decide whether to take part. While participants who received information through practitioners were known not to meet exclusion criteria, families approached by the local charity were asked to have a consultation with one of the collaborating practitioners to ensure their child did not meet any exclusion criterion. Parents who agreed for their child to take part provided written consent before starting the study.

### Interventions

Infants in the ACT intervention watched interactive cartoons on a computer screen connected to an eye-tracker which recorded the infant’s eye movements: the computer ran a software that produced the animations on the screen (see ‘Equipment and material’). We used three type of tasks that trained key abilities such as the following: search for a target among distracters (three games—‘Stars’, ‘Usual Suspects’ and ‘Disengagement’); short-term memory of objects embedded in scenes (‘Puzzle Memory’, ‘Windows’, ‘Tausendfuss’ and ‘Three Little Maids’); maintaining a goal (‘Butterfly’ and ‘FlyMe’). Further details on these tasks are provided in the published protocol [50] and the Supplementary Material.

The types of tasks presented were spread across the three categories and delivered according to a computerised random order. The experimenter was instructed to present each game for at least 240 s without interruptions. Training games could be interrupted if the child became sleepy or irritable: games were interrupted when infants failed to engage with the game for a continuous period of over 30 s. A web camera mounted above the screen displaying the cartoons recorded the infant’s face: this was used in order to check quality of data produced and to complement potential loss of data.

The control procedure prescribed presenting cartoons in the same way as in the ‘Interventions’ and with the same equipment. The key difference was the type of presentation on the screen: in the control procedure, these were not interactive and thus did not change contingently with infants’ gaze direction. To ensure presentations were similar in length and characteristics to those in the intervention group, infants in the control group were matched infant-by-infant and visit-by-visit with participants in the ACT training. Thus, the cartoons displayed to a control child were the same ones produced by the corresponding matched child in the intervention group: The pivotal difference was that the display was generated according to a pre-set schedule for those in the control group.

Infants watched the training or control games while sitting on the parent’s lap, approximately 40 cm from the screen. The screen, the parent and the infant were inside a cubic photo light tent to avoid visual distractions and sudden changes in light.

The experimenter introduced the training tasks to parents describing them as ‘games’ and stating the aim was for infants to play the games for as long as possible. The experimenter also explained that the eye-tracker had a limited ‘head box’, i.e. an area within which the devise can detect the infant’s eyes: parents were asked to try to keep the baby’s head within this headbox. The experimenter told parents they should try to re-engage their child’s attention in the direction of the screen if she turned away, but to do so by using non-descriptive phrases such as ‘look’. Otherwise, parents were encouraged not to talk or to do anything that could distract the child while the games were running. The experimenter was instructed to monitor the parent and infant’s behaviour through the web camera, and if necessary remind parents about these instructions.

The training and the control sessions could take place in a dedicated room in the charity’s premises, or at the parents’ home, according to parent’s preferences. When conducting the sessions in the family’s home, researchers asked for collaboration in minimising any other potential source of distraction, e.g. loud or sudden noises. To monitor differences in conditions across settings, a coder (author MJ) reviewed the recordings of the visits, rating the presence of noise and light interference in a Likert-type scale (from *no interference* to *major interference*) and the infant’s state (drowsy, calm and alert, mild protest, distressed or crying). The main author reviewed a sample of these sessions (30%) to check inter-rater reliability, obtaining adequate indices (Cohen’s *κ* > .75 for all categories).

### Equipment and material

The games were presented on a standard 19” 4:3 computer screen. Stimuli presentations were controlled by an Apple MacBook Retina running Matlab scripts, operating via Psychtoolbox/GStreamer and the Tobii SDK. A Tobii X-60 eye-tracker mounted at the bottom of the screen fed information to the laptop, after an initial calibration sequence. Matlab also recorded the eye-tracker gaze data.

### Outcomes

- *Recruitment*: Recruitment was defined as the percentage of the eligible families approached who agreed to take part in the study and were randomised.
- Retention: This was the percentage of randomised participants for whom data were available at baseline and post-test.
- Number, percentage and duration of training/control sessions attended and completed by infants.
- We also assessed changes in performance of trained infants during the training: improved performance would indicate infants were engaging with the training and gaining proficiency.
- Percentage and type of tasks for which data are available at pre- and post-test.
- Quality of eye-tracker data collected during baseline and post-test assessments, such as the number of usable fragments and the degree of consistency in the reported position of gaze between recorded samples (see ‘Statistical analysis’).

We also collected feedback from participating parents using a short questionnaire and a semi-structured interview. In the latter, parents were asked about difficulties and obstacles in taking part and remaining in the study, as well as their motivations in taking part and their experience of the study.

### Baseline and post-test assessments

We used a battery of tests to gather information about the infants’ general cognitive and motor development, their attention and their social cognition abilities. The presentation of tasks took place in four pseudo-randomised sequences, counterbalanced between infants and across baseline and post-test within each infant. At baseline, parents also completed a questionnaire to collect socio-demographic information about them (e.g. educational attainment) and their child (e.g. birth weight). We describe these tasks to illustrate the commitment required by infants and families and their ability to complete these, the main aim of this study. Further analyses of task-specific results will be presented in another paper.

#### Setting of baseline and post-test assessments

The battery of tasks took place in a dedicated room in the premise of the participating charity. The room had no windows and contained the photo light tent used for the eye-tracking attention tasks, as well as a desk and two chairs where all the tasks not requiring eye-tracking data were delivered. Two CCTV cameras were placed at opposite angles around the desk in order to obtain a simultaneous recording of the infant and the parent, and the assessor. In most of the desk-based tasks, the assessor sat on one side, and the infant sat on the parent’s lap, opposite the assessor.

#### General cognitive and sensory-motor development

We administered the Mullen Scales of Early Learning [44]. These provide scores on different domains of infants’ development (e.g. expressive language; fine motor abilities), including norms. Results at baseline are reported in Table 1.

**Table 1:** Participant characteristics by intervention and control groups

	Intervention				Control			
	**Full-time employment**	**Part-time employment**	**Not in employment**	**Information not provided**	**Full-time employment**	**Part-time employment**	**Not in employment**	**Information not provided**
Mother’s employment	3 (50.00)	2 (33.33)	0 (–)	1 (16.67)	2 (33.33)	2 (33.33)	2 (33.33)	0 (–)
Father’s employment	4 (66.67)	1 (16.67)	0 (–)	1 (16.67)	4 (66.67)	0 (–)	1 (16.67)	1 (16.67)
	**Degree**	**A-level**	**GCSEs**	**Information not provided**	**Degree**	**A-level**	**GCSEs**	**Information not provided**
Highest mother’s educational attainment	5 (83.33)	0 (–)	0 (–)	1 (16.67)	3 (50.00)	1 (16.67)	1 (16.67)	1 (16.67)
	**Females**	**Males**	**Total**		**Females**	**Males**	**Total**	
Infant’s sex	2 (33.33)	4 (66.67)	6		2 (33.33)	4 (66.67)	6	
	**Mean**	**SD**	**Total**		**Mean**	**SD**	**Total**	
Gestational age (weeks)	29.2	1.09	5		30	1.09	6	
Birth weight (g)	1313.25	338.46	5		1378.60	332.13	6	
Days in NICU	57.6	20.95	5		69.8	62.83	5	
APGAR (5 min)	4.33	4.16	3		8.5	0.71	2	
Age (months): pre-test	11.90	0.79	5		13.04	1.21	6	
Age (months): post-test	12.95	0.78	5		14.43	1.01	5	
Mullen cognitive T scores at pre-test	94.40	11.46	5		92.67	14.85	6	

#### Computer-based measures of attention

We delivered widely used measures of sustained attention, visual recognition memory, disengagement and information processing. These took place inside the photographer tent, while the infant sat on the parent’s lap in front of a computer screen. The tasks are described in the study protocol [50].

#### Naturalistic attention tasks

We used the Orientation Task from the Lab-Tab [31, 51], as well as a semi-structured interaction between parent and infant. In the latter, four age-appropriate attractive toys were placed in front of the infant and the parent sat across the table. Parents were instructed to play with the infant as they ‘would normally at home’ for 4 min.

#### Social attention and cognition

We administered the Gaze Following, Object Spectacle and Book Presentation tasks from the Early Social Communication Scales (ESCS) [45]. These provide information on the infant’s ability to share attention with other people and her communicative abilities.

#### Temperament

Information on some temperamental traits is provided by the Orientation Task mentioned among the naturalistic attention tasks. We also administered the Attractive Toy Placed in a Box task from the Lab-Tab [31, 51]. The latter provides measures relating to behaviour regulation. Furthermore, parents were also asked to complete the very-short form of the Infant Behaviour Questionnaire (IBQ) [52].

### Sample size

The intended sample size for the study had been determined using a confidence interval approach to power calculation [19]: our aim was to recruit 20 infants.

### Randomisation

#### Sequence generation

The random allocation sequence was created in two blocks (*n* = 10 each) using randomly generated numbers from the uniform distribution. These were generated using Stata 13 [65] and constraining the intervention/control ratio to be 1:1.

#### Allocation concealment mechanism

The experimenter responsible for delivering of the intervention/control received the allocation in a sealed opaque envelope when a new participant was due to commence testing. The experimenter opened the envelope only when the child finished the baseline assessment and was instructed not to disclose allocation to the assessor responsible for delivery of the post-test.

#### Implementation

The sequence generation was produced by the first author and project PI. Participants were enrolled by the experimenter, who did not administer the baseline and post-test outcome measures, but during these tasks attended to the recording cameras.

#### Blinding

To ensure blinding, the assessor did not contribute to administration of the training/control procedures, nor were they involved in data analyses concerning training and control procedures. We informed parents that we were not going to tell them in which group their child had been allocated. Parents were thus intended to remain blind to group allocation. Since the parents were present during the training or control sessions, they might identify whether the games were part of the training (i.e. they were interactive displays), or the control procedure (i.e. games were not interactive). Our experience suggested it was unlikely parents recognised the infant’s group allocation since infants in both conditions react to the displays with signs of interest, due the attention-grabbing nature of the stimuli (e.g. moving high-contrast stimuli). Furthermore, parents could not see the eyes of their infant while the infant was on their lap, and therefore, it might have been difficult for them to assess if the cartoons were responding contingently to the infant’s eye direction. However, at the end of the study, we asked parents to indicate whether they thought they had recognised to which study arm the child had been allocated, and in which study arm they thought their child was.

### Ethical approval

The study had been reviewed and approved by the Health and Social Care Research Ethics Committee A (HSC REC A), REC reference: 18/NI/0010; IRAS project ID: 37537. The original protocol was changed to allow researchers to ask parents to take part in a phone interview at the end of the study and relate their experience of participating in it. Parents who agreed to take part in this interview expressed their informed consent recorded before commencing the interview. The protocol was also changed to allow families to opt to carry out the intervention or control procedure in their own home.

### Statistical analysis

#### Recruitment and retention

We used descriptive statistics to indicate the number and proportion of participants recruited relative to eligible and approached individuals, and the absolute and relative numbers of participants retained and their characteristics.

#### Duration and completion of training and control session

The duration of training and control sessions was reported in minutes. Descriptive statistics were used to report the average number and duration of training or control tasks by sessions, as well as cumulatively across the study. Further analyses indicated the number and duration of training tasks by type of tasks (goal maintenance, target search, short-term memory).

#### Performance during training tasks

Performance indicators varied according to tasks’ demands and criteria (see Supplementary Material). Performance indicators were standardised into *Z* scores to ease comparisons across tasks. We then collated individual participants’ Z scores across tasks of the same type (goal maintenance, target search, short-term memory) in each training visit: thus, each infant contributed several performance measurements during each visit. We used multilevel growth regression models to test if trained infants displayed linear changes in performance across visits: linear effects would indicate significant performance improvement across tasks. In these analyses, the units of observations were the *Z* scores registered across visits, which were considered nested within infants. The models allowed to estimate the *initial status* (average *Z* scores in the first visit) and the *rate of change* (the average change in performance *Z* scores from one visit to another), as well as residual variances around these parameters (see Supplementary Material). Multilevel growth models allow to control for inter-individual differences and can also control for the fact that participants contribute different number of observations: multilevel models are thus ideally suited for analyses of our data.

#### Completion of pre- and post-test assessment tasks

We used descriptive statistics to report on the number of pre- and post-assessment tasks completed by group.

#### Quality of eye-tracking data

The quality of eye-tracking data recorded was assessed in the gap-overlap task of all participants at pre- and post-test. We chose this task because, in our experience, it is generally the most attractive to infants and they tend to engage in this task more than others. Thus, the task provides a meaningful sample of the eye-tracker data quality; insofar, the recording should be less affected by infants’ losing interest and disengaging from the task.

Following methods described by Wass et al. [77], we calculated two indices: *precision* of tracking, and *flicker*, which is an indicator of the robustness of the recording.

Precision of tracking indicated variability in the degree to which the position of gaze was consistent between recorded samples. Increased variability in the recording of the position of gaze from one gaze sample to the next would indicate increased sampling error and a ‘noisier signal’. Thus, more precise recordings are characterised by lower degree of variation in position of gaze reported.

Flicker was defined as the duration in seconds of usable fragments of eye-tracking data during the task recording. The loss of usable fragments during a recording can be caused by unavailability of any of the elements the eye-tracker uses to assess gaze direction (e.g. when infants turn away from the screen). Thus, a longer duration indicated more robust recordings.

These two indicators of data quality are important insofar as lower-quality data can lead to unwarranted conclusions when comparing performance of different groups (e.g. typically vs. at-risk infants): for example, worse quality of data can bias reaction times to appear shorter (see [77]). To gauge the quality of data obtained from our sample of VP infants, we compared the values of Precision and Flicker with the values obtained in the study by Ballieux et al. [6] and the study by Wass et al. [74]. Both these studies involved typically developing infants born at term, but Ballieux et al. conducted their assessments in a setting more akin to that used in this study (a day centre), while Wass et al. conducted their assessments in a laboratory.

#### Qualitative outcomes

Follow-up semi-structured interviews with parents that had taken part in the study were conducted on the phone by the first author, who followed an interview schedule. These were transcribed verbatim and analysed using thematic analysis to identify common themes related by participating parents. These analyses were conducted by a researcher not involved in the project who independently identified themes. The first author reviewed the transcripts to ascertain validity of interpretation.

## Results

### Recruitment

**Fig. 1:**
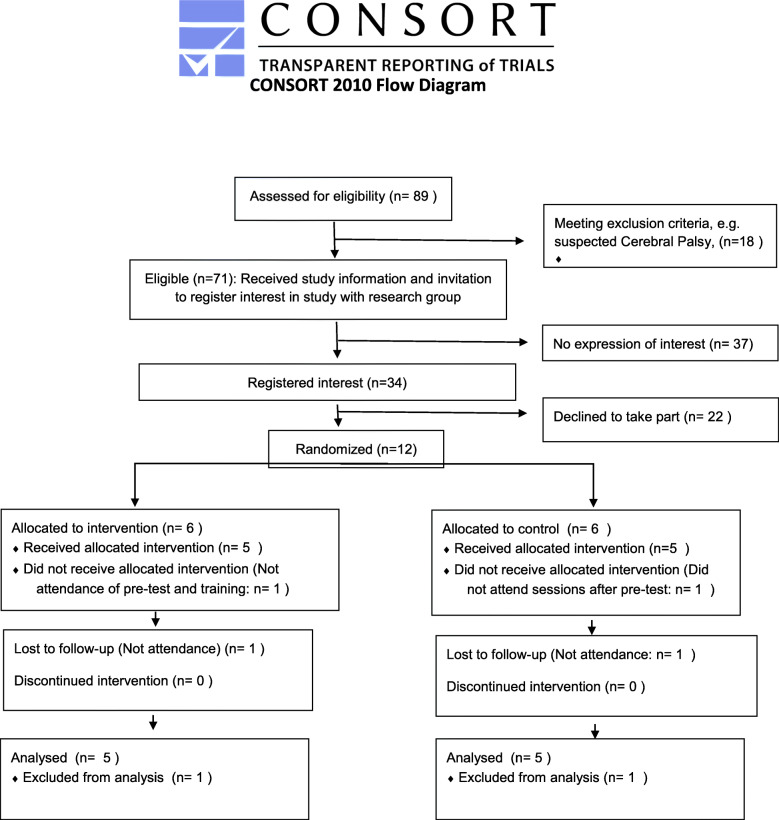
CONSORT diagram

The recruitment phase encompassed 13 months from April 2018 to April 2019. Results are reported using the CONSORT flow diagram (Fig. 1). Overall, of all the families eligible and contacted by the gatekeepers, 27 recorded their interest for the study by contacting the research group: a large proportion of these thus failed to register their interest in the study after receiving information through the gatekeepers. Seven of these families had twins, thus representing 34 eligible participants that expressed interest in the study. Out of these, 11 families agreed to take part (41% of those who recorded their interest). These accounted for 12 infants being randomised to take part in the study, as one family had twins.

Information on infants and their families was available for 11 infants who attended the first session with their families. The primary caregiver of infants who took part in the study was the biological mother. The highest educational attainment for most of them (73%) was a university degree. Furthermore, 7 mothers reported attainment of A-levels, and 8 reported attainment of GSCEs, which are the minimal educational attainments expected in the UK. Overall, participating families had parents with higher educational attainments than average. Families whereby mothers were in full-time employment also had partners in full-time employment: only one family reported both parents were not in paid employment. Parents’ and infants’ characteristics are reported in Table 1.

### Retention

Out of the 12 infants randomised in the study, 10 (83%) were retained (see Fig. 1). These 10 completed the tasks in the baseline and post-test assessments (see section on ‘Completion of pre- and post-test assessment tasks’, for more details). One infant, who had been randomised to the intervention, did not attend the baseline assessment and our attempts to reschedule other appointments were unsuccessful. Another infant completed the baseline assessment and was randomised to the control group but did not attend the visits afterwards. The 10 infants that completed the study were evenly split between the intervention and control groups.

**Fig. 2:**
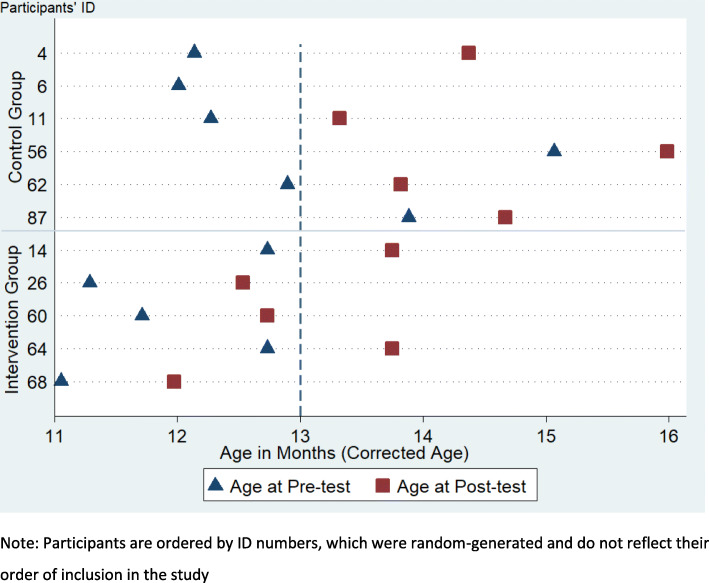
Age (in months) at pre- and post-test of participants. Each row represents a participant, with triangles indicating the age at pre-test and the squares representing the age at post-test. The dashed line represents the threshold of 13 months of age, within which we had intended to run participants’ pre-test. Note: Participants are ordered by ID numbers, which were random-generated and do not reflect their order of inclusion in the study

In Fig. 2, we report the corrected age (CA) in months when infants attended the pre- and post-test sessions: these are calculated based on infant’s gestation age and expected date of birth reported by parents. The average age at pre-test was 11.90 months CA (*SD* = 0.79) and 13.04 months CA (*SD* = 1.21) for the intervention and control groups respectively. The average age at post-test was 12.95 months CA (SD = 0.78) and 14.43 months CA (SD = 1.01) for the intervention and control group respectively. The families of two infants in the control group agreed to take part in the study and had scheduled an appointment to take place by the time the infants were 12 months CA, but because of family commitments were only able to attend the pre-test assessment when aged over 13 months CA. Family commitments also caused delays in completing the post-test of ID 4 (control group), who was approximately 12 months CA at pre-test and was over 14 months CA when completing the post-test. Since differences in age and maturation have an impact on attention control, these differences across participants and across groups must be taken into account: in future data analyses, we will have to either control for age effects or exclude participants tested outside the range of 12 months (± 1 month). Overall, results indicate some difficulties in scheduling appointments within the intended age range of our study.

### Number, duration and characteristics of training/control sessions completed

All infants in the training and the control group attended the visits scheduled in weeks 2 to 4 of the procedure. Furthermore, two infants in the intervention group and one in the control group also started their training/control procedure on week 1, after they completed the pre-test assessments. Thus, the 10 participants cumulatively completed 33 visits, comprising 17 intervention and 16 control completed visits respectively. Overall, 2 out of 17 intervention visits, and 6 out of the 16 control sessions had been completed at the participants’ home: only one infant in the intervention group received the training at home (and only for 2 out of 3 sessions), and two infants in the control group completed the control procedure at home.

Participants in the intervention group completed 4.65 games per session (SD = 1.62), see Table 2. For participants in the intervention group, a game was deemed completed if the infant engaged in it for at least 240 s: in previous studies using the ACT, this had been identified as an adequate length of engagement with training games. The average percentage of games completed was 73.16% (SD = 26.63). The average duration of completed games per visit in the intervention group was 24.59 min (SD = 9.39). Participants in the intervention group cumulatively completed 83.60 min of training games per child, with a range between 65.95 and 104.05 min.

**Table 2:** Number of games completed, proportion of completions for the intervention group and duration of sessions completed by group allocation and by session

		***N*** games completed		Proportion games completed		Duration games completed in minutes	
	*Group*	*Average*	*SD*	*Average*	*SD*	*Average*	*SD*
After pre-test	Intervention	1.00	1.41	0.25	0.35	4.71	6.66
	Control	3.00		–	–	12.71	
Week 1	Intervention	5.00	1.22	0.74	0.21	24.91	6.55
	Control	4.20	2.39	–	–	17.45	9.64
Week 2	Intervention	5.20	0.45	0.79	0.16	26.92	2.67
	Control	5.60	0.55	–	–	24.16	4.88
Week 3	Intervention	5.20	0.84	0.86	0.20	29.88	7.20
	Control	6.20	0.45	–	–	30.92	5.89
**Overall**	**Intervention**	**4.65**	**1.62**	**0.73**	**0.27**	**24.59**	**9.39**
	**Control**	**5.19**	**1.64**	–	–	**23.46**	**8.88**

The cumulative training time of 84 min was remarkably similar to the 77 min of cumulative training time displayed by typically developing infants in the study by Wass and colleagues [74], which prescribed the same number of training/control sessions as our study. Infants in the intervention group completed an average of 25 min of training (respectively 5, 25, 27, and 30 min from the first visit following pre-test to the last visit): these times were also very similar to those reported in another study involving typically developing infants [6] who on average completed 10, 21, 19, and 25 min of training from the first to the last visit, respectively.

The average duration of the games displayed to participants in the control group was 23.46 min (SD = 8.88). Overall, the five participants in the control group attended 75.07 min of games per child, with a range between 61.41 and 87.88 min. The procedure of displaying to controls the training sessions of a matched trained infant should have enabled identical duration of sessions for the control group: instead, the results indicated a relatively shorter cumulative duration of the control sessions. This can be partly explained by the contingent fact that one control child completed only a few minutes of the procedure in week 1 (as reflected by lower average duration for the controls in week 1, see Table 2). However, the average duration of training and control sessions was similar (24.59 and 23.46 min respectively).

The two training visits delivered at home to one participant had an average duration of 26.99 min (SD = 2.72), which compared favourably with the average durations of training visits in the lab across participants (mean = 24.27 min; SD = 9.96). Across control participants, the average duration of the visits at home and in the lab was 21.61 min (SD = 10.04) and 24.57 min (SD = 8.47) respectively.

**Fig. 3:**
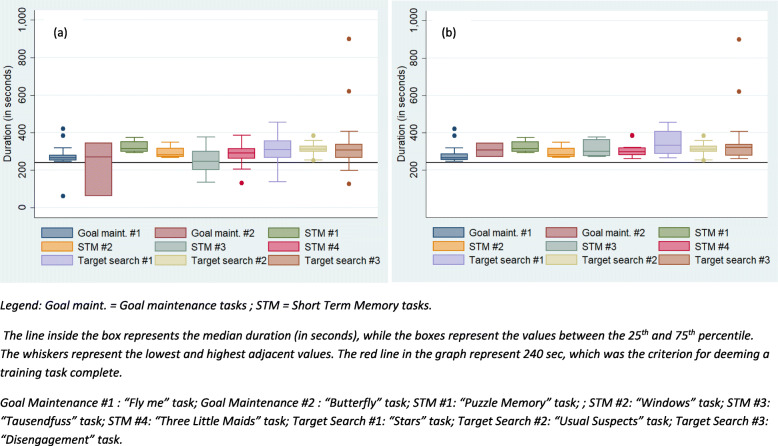
Boxplots of task duration in the intervention group (*n* = 5) over training sessions. **a** Boxplots of complete and incomplete tasks. **b** Boxplots for completed tasks only (i.e. those lasting at least 240 s). Legend: Goal maint. = goal maintenance tasks; STM = short-term memory tasks. The line inside the box represents the median duration (in seconds), while the boxes represent the values between the 25th and 75th percentile. The whiskers represent the lowest and highest adjacent values. The red line in the graph represents 240 s, which was the criterion for deeming a training task complete. Goal maintenance #1: ‘Fly me’ task; Goal maintenance #2: ‘Butterfly’ task; STM #1: ‘Puzzle Memory’ task; STM #2: ‘Windows’ task; STM #3: ‘Tausendfuss’ task; STM #4: ‘Three Little Maids’ task; Target search #1: ‘Stars’ task; Target search #2: ‘Usual Suspects’ task; Target search #3: ‘Disengagement’ task

The median duration of each training game presented to the intervention group are reported in Fig. 3a: these summarise durations of completed and attempted presentations for each game. Despite some variability (e.g. the duration of the goal maintenance task #2, due partly to some technical problems with this task), all the median durations were above the 240 s threshold. Figure 3b presents the median duration of the completed games (i.e. those lasting at least 240 s): overall, participants displayed adequate durations of engagement across all the tasks. In Table 3, we also report the cumulative number and duration of completed games by type of games. Participants engaged for adequate amount of times with all types of tasks. It is notable that every child across the study completed at least three tasks within each of these categories, indicating that they received training of all the three abilities targeted.

**Table 3:** Cumulative number and duration of completed tasks by type of task of the intervention group (*n* = 5)

	Search for target		Short-term memory		Goal maintenance	
	*N* of tasks	Duration in minutes	*N* of tasks	Duration in minutes	*N* of tasks	Duration in minutes
Average	6.8	38.55	5.4	27.90	3.6	17.15
SD	0.84	9.44	2.07	9.26	0.55	4.03
Min	6	28.87	3	16.48	3	12.77
Max	8	54.25	8	39.20	4	21.13

The training or control visits delivered in the home setting were overall rated to take place in adequate conditions. Approximately 2% of the tasks delivered at the participants’ home were considered to take place while some acoustic distraction was present and when abrupt changes in light took place, which might have interfered with the task delivery. In approximately 89% of the tasks, the participating infant was considered in an alert and calm state.

### Performance during training

**Fig. 4:**
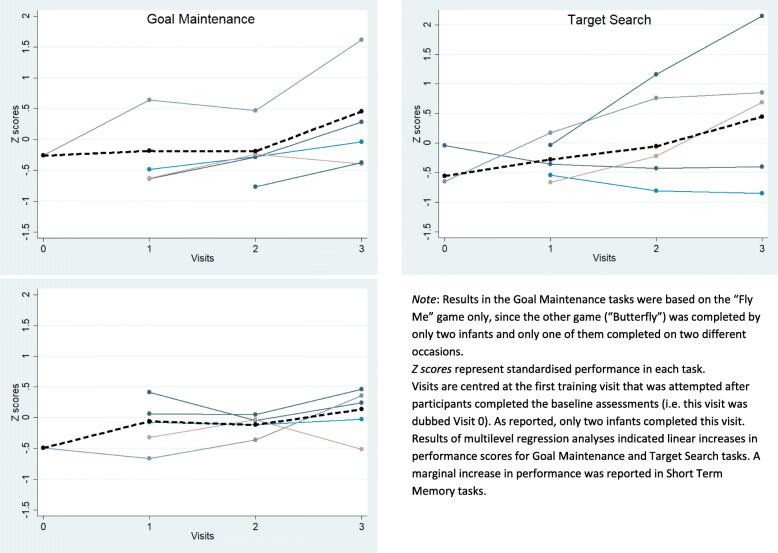
Performance in the three types of games by visit. The dashed lines represent average across all infants in the training group, while the full lines represent participants’ individual averages

In Fig. 4, we report standardised (*Z*) performance scores across the three types of tasks delivered, while details about the model parameters and results are reported in the Supplementary Material. A multilevel growth model indicated that infants displayed a relevant linear increase in performance in the goal maintenance task, rate of change coefficient 0.41 (*95% CI* 0.23 to 0.59; *z* = 4.50). Infants also displayed a linear increase in Target Search performance across visits, rate of change coefficient 0.40 (*95% CI* 0.04 to 0.76; *z* = 2.20). Finally, multilevel regression indicated a marginal linear increase in short-term memory performance across visits, rate of change coefficient 0.12 (*95% CI* − 0.01 to 0.25; *z* = 1.79). Overall, these results indicated that infants improved performance across training visits, suggesting adequate engagement with the training.

### Percentage and type of tasks for which data are available at pre- and post-test

The completion of the test battery devised took between 42 and 99 min to complete, with an average of 66.62 min (*SD* = 16.51). All the participants that attended the pre-test sessions in the intervention and control groups (*n* = 5 and *n* = 6 respectively) completed the screen-based attention tasks; all those that attended the post-test in the intervention and control group (*n* = 5 in both groups) completed the screen-based attention tasks.

We also recorded good rates of completion of social attention and cognition tasks from the ESCS, as reported in Table 4. Overall, all the tasks were completed by participants from both groups who attended the pre-test. Only one participant in the intervention group failed to complete both the Gaze Following and the Object Spectacle tasks during the post-test because of tiredness. While non-completion of these tasks because of tiredness may signal obstacles that may be experienced by other participants, it should be taken into account that this participant was from a couple of twins enrolled and being tested on the same day: The participant was randomly chosen to be tested after the other twin, therefore remaining in the premises where we conducted testing for longer, compared to other participants.

**Table 4:** Percentages of participants completing the ESCS tasks and percentages of trials completed within each task. Gaze Following involved 4 trials; Book Presentation and Object Spectacle 6 trials each

		Gaze following				Book presentation				Object spectacle			
		Participants completing task		Proportion of tasks completed		Participants completing task		Proportion of tasks completed		Participants completing task		Proportion of tasks completed	
		*Pre-*	*Post-*	*Pre-*	*Post-*	*Pre-*	*Post-*	*Pre-*	*Post-*	*Pre-*	*Post-*	*Pre-*	*Post-*
Intervention	Average	100.0%	80.0%	100.0%	80.0%	100.0%	100.0%	90.0%	80.0%	100.0%	80.0%	90.0%	80.0%
	*SD*	–	44.7%	–	44.7%	–	–	14.9%	21.7%	–	44.7%	22.4%	44.7%
Control	Average	100.0%	100.0%	100.0%	100.0%	100.0%	100.0%	91.7%	93.3%	100.0%	100.0%	100.0%	100.0%
	*SD*	–	–	–	–	–	–	13.9%	9.1%	–	–	–	–

All participants who attended the pre- and post-test completed the Mullen Scales of Early Learning, as well as the as well as the naturalistic attention tasks: the Lab-Tab Orienting task and the semi-structured parent-infant interaction, although the latter had not been recorded at post-test for one child from the control group because of an error using the recording equipment. All infants that attended the experimental sessions also completed the Lab-Tab Frustration task, and the IBQ questionnaires were returned by parents for all infants at pre- and post-test, save for the parents of an infant in the control group that did not return the questionnaire at post-test. Overall, the results indicate that the battery of tasks we administered was feasible.

### Quality of eye-tracker data collected during baseline and post-test assessments

We used two indicators recorded during the pre- and post-test gap-overlap task: variability in Precision and Flicker. The Standard Error of the Mean (SEM) of the variability in Precision obtained from 21 completed gap-overlap tasks at pre- and post-test was 6.2e−03 s (*SD =* .6e−03 s, range 3.0e−03 s to 13.9e−0.3 s). This was a relatively higher value compared to similar studies that reported *SEM =* 4.0e−03 s and SD = .2e−03 s [6] and *SEM =* 3.2e−03 s and SD = .1e−03 s [74] respectively, indicating less precise tracking overall.

Flicker was indexed by values indicating continuous recording, whereby higher values indicate fewer instances of information loss. The SEM of Flicker recorded across 21 completed tasks in our sample was 1.51 s (*SD =* 0.24 s; range 0.47 to 4.88 s). The SEM of this indicator was lower compared to that obtained in other similar studies: SEM *=* 2.0 s and SD *=* .18 s [6]; SEM *=* 3.7 s and SD *=* .18 s [74], respectively, indicating less robust tracking.

### Feedback from parents

#### End-of-study questionnaire

Seven parents completed the end-of-study questionnaire, accounting for 8 participating infants (5 of whom allocated to the intervention). In Table 5, we report summary statistics of parents’ answers: scores ranged from 5 (strongly agree) to 1 (strongly disagree), so that higher scores indicate agreement with the question statement.

**Table 5:** Average and SD of parents’ responses to the end-of-study questionnaire. Scores ranged from 1 to 5, whereby 1 indicated non-agreement with a statement (i.e. ‘strongly disagree’), and 5 indicated agreement with the statement (i.e. ‘strongly agree’)

		Written information clear	Oral communication clear	Difficulties understanding information about study	The study was important	Difficult to attend weekly	Would have preferred home visits	Sessions too long	Baby enjoyed study	Baby tired by end of sessions	Taking part demanding	Would recommend to other parents	Would take part again	Would have liked to know group allocation
Control	Mean	4.67	4.67	1.00	4.67	1.00	2.33	2.00	4.00	4.00	1.33	4.67	5.00	2.67
	*SD*	*0.58*	*0.58*	–	*0.58*	–	*1.15*	*1.00*	–	*1.00*	*0.58*	*0.58*	–	*0.58*
Intervention	Mean	4.75	4.75	2.25	5.00	2.25	3.33	2.25	3.75	3.75	2.00	4.75	4.75	2.75
	*SD*	*0.50*	*0.50*	*1.89*	*0.00*	*1.26*	*0.58*	*0.96*	*0.50*	*0.50*	*1.41*	*0.50*	*0.50*	*0.50*
Total	Mean	4.71	4.71	1.71	4.86	1.71	2.83	2.14	3.86	3.86	1.71	4.71	4.86	2.71
	*SD*	*0.49*	*0.49*	*1.50*	*0.38*	*1.11*	*0.98*	*0.90*	*0.38*	*0.69*	*1.11*	*0.49*	*0.38*	*0.49*

**Fig. 5:**
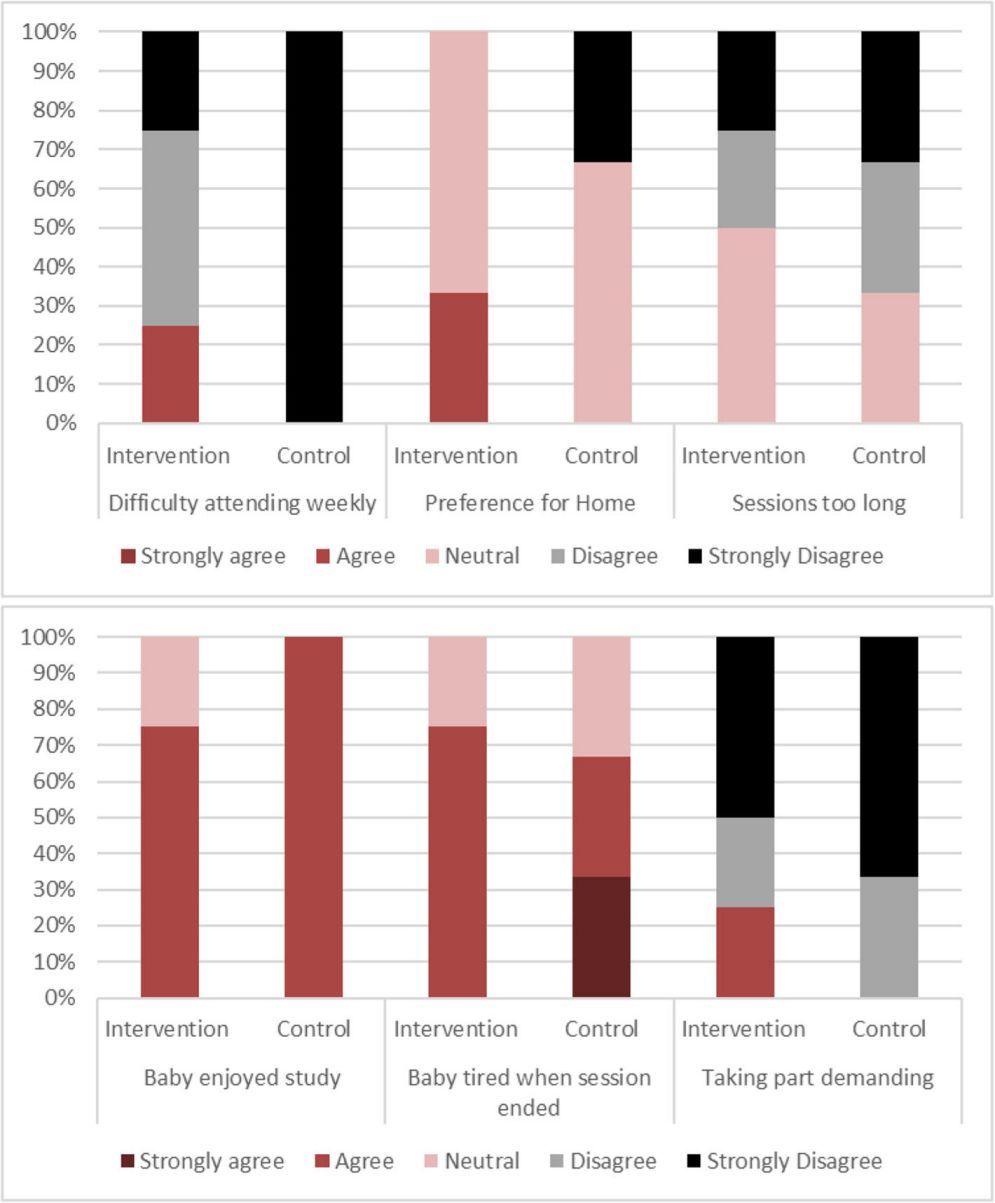
Parent’s responses to questions on obstacles to participation by group allocation

Parents generally agreed that the information provided and the communication by the research team was clear, were convinced about the importance of the study, and would recommend taking part to other parents. In Fig. 5, we report a more detailed breakdown of the questions that concerned obstacles in study participation and the infants’ perceived experience. These results indicate some parents across the two groups found the study sessions too long, and all parents generally agreed that infants were tired at the end of these sessions. However, only one parent indicated taking part was demanding. Nonetheless, all parents who replied agreed their infants had enjoyed taking part in the study.

Results also indicated that parents generally accepted the process of randomisation: the need for random allocation was explained in the information sheet and parents required to agree to this process. Furthermore, parents did not express the desire to know in which group had been allocated, see Table 5. Finally, parents correctly identified the group allocation of their child in 4 out of 8 instances.

#### Semi-structured interviews

Five parents from five different households of infants who took part in the study were available to conduct an interview at the end of the study. Three of these had infants who had been allocated to the intervention, and two completed the study before the option of home visits had been offered. The themes identified are summarised in Table 6.

**Table 6:** Themes and sub-themes identified by the thematic analysis of interviews with parents of infants who took part in the study

Decision to take part	**Proximal:** Contact by known gatekeeper (consultant or charity); Sense of gratitude towards the gatekeeper; Clarity of communication by research team. **Distal:** Desire to help other families and preterm babies; Interest in infants’ cognitive development.
Facilitators	**Extrinsic:** Staff being flexible and approachable; Regular reminders from staff; Manageable schedule; Sessions conducted at home; **Intrinsic:** Assessing baby’s progress; Baby enjoying the tasks
Obstacles	Fitting lab visits with baby’s routines, as well as other practical challenges (e.g. arranging siblings’ child care; work patterns). Baby’s sleep patterns and moods; Maintaining infants’ concentration
Improvements	Provision of exercise before long sitting sessions; Provide meals or refreshments; Minimise concerns about potential for infections. More specific information about duration of training tasks.

The first topic concerned the decision to take part in the study. Our analyses identified some ‘proximal’ factors that concerned their relationship with the gatekeepers who approached the family in the first instance. All respondents emphasised trust and a sense of gratitude towards the gatekeeper. For example:

‘It was because of the consultant ringing, because we have a relationship with him, and he was very very good with [baby].’ [Father participant ID 14, intervention group].

‘It was just what I could do to give back really: we had got quite a bit of support from TinyLife, so because it was through them, we were like: Oh yeah, will give it a go.’ [Mother participant ID 11, control group].

Parents also discussed other more general, ‘distal’ factors as important in their decision to take part. These included the desire to potentially help families and preterm infants by contributing to research, as well as interest in learning more about infants’ cognitive development.

Respondents discussed a series of factors that facilitated their and their child’s participation in the study. Some were ‘extrinsic’ insofar they involved research staff behaviour and contextual factors, such as the study schedule. All respondents appreciated researchers being approachable and providing flexibility in scheduling appointments and valued the opportunity to run the training/control games in their own home. Some parents also commented positively on the study schedule, for example:

‘The fact it was only once a week worked well because I have a busy schedule anyway with the baby so, but I think if it was more than once a week it, you know, would be hard to fit into our schedule.’ [Mother participant ID 60, intervention group].

Other factors that facilitated participation in the study were considered to be ‘intrinsic’, i.e. they concerned the fact that infants engaged and enjoyed some of the tasks, and the satisfaction of being able to observe baby’s progress across time. For example:

‘[…] Just even for my own benefit to see that [baby] was progressing and, you know, learning as the weeks went on, and by the end of it was able to do stuff he couldn’t at the beginning. Haha, you know, that was probably the best part of it for us, seeing the progress.’ [Mother participant ID 11, control group].

Respondents reported a series of difficulties in participating to the study. Parents commented on the challenges of fitting study attendance with infants’ routines (e.g. meal and sleep times), and they also commented on the challenges related to travel to attend study visits while accommodating infants’ feeding and sleep patterns. Some also commented on difficulties related to infants’ mood being ‘unpredictable’, and the challenges provided by attempting to maintain infants’ concentration for the length of the study games and tasks.

Finally, parents mentioned some potential incentives and facilitators to the study procedure. One parent (infant ID 14) suggested the possibility of providing light meals and refreshments to facilitate participation of families, particularly if testing took place outside of normal working hours. Other issues concerned the potential benefit of providing some physical activity for infants before they were expected to sit in front of the screen during the testing/control games (Parent of infant ID 60). One parent also emphasised the importance of providing a testing environment that was safe from dangers of infection that may derive, for example, from infants sharing the same toy. Finally, some comments concerned the provision of more detailed information concerning the length of the testing/control games:

‘If there was some way for the parent to have an idea of how each clip was going to run for. Because obviously we’re trying to sit and hold them in place, it would be good to know how long they’re going to last and when it’s going to end.’ [Father participant ID 14, intervention group].

## Discussion

We conducted a study to investigate the feasibility of delivering a computerised cognitive training intervention, the Attention Control Training (ACT), to infants born very preterm. Our overarching aim was to test the processes to be used in a randomised trial, including recruitment, retention, treatment adherence and acceptability of the outcome measures. We had set a priori criteria for assessing feasibility (see ‘Background’) that were mostly met. In particular, (a) we had aimed to recruit 20 VP infants within a year, but managed to recruit 12, thus failing to meet this criterion; (b) we considered successful retention if 80% of recruited infants completed the study: 10 out 12 (83%) did so according to the criteria set (see point *d* below); (c) we considered success in the procedure if infants retained completed at least two tasks for 240 s on at least two weekly session: the 10 infants retained in this study met this criterion (see Table 2 and ‘Results’ section); (d) we considered the battery of pre- and post-test assessments feasible if retained infants completed at least 50% of these tasks: all 10 infants retained met and exceeded this criterion (see Table 4 and ‘Results’ section). The results therefore indicate the study is feasible overall, but also emphasise key challenges in recruitment. In what follows, we will map our results onto the study questions.

### Recruitment

The results indicate that while it is possible to recruit families of very preterm infants, there are challenges that still need to be addressed. We had aimed to recruit 20 infants, but recruited 12 infants from 11 households. Furthermore, most participants came from households where the main caregiver reported higher than average educational attainments. Improving recruitment rates would also necessitate recruiting families with diverse backgrounds.

VP infants and their families represent a relatively small and hard-to-reach population. Vulnerability to health problems and differences in temperament and attention, which are evident from an early age, may hinder VP infants’ participation in cognitive training programmes like the ACT. Furthermore, parents may also be affected by a VP birth by displaying increased concerns, anxiety and mental health issues [69]. Recruitment should thus be tailored to take into account these issues.

Our recruitment process relied on two types of gatekeeper to inform eligible participants about the study: practitioners known to the family, and a dedicated charity. The findings of the qualitative analyses of participants’ feedback suggest that this strategy was effective: parents reported that one of the drivers in their decision to take part in the study was trust and their personal relationship with the gatekeeper. Furthermore, charity workers promoted the study, a strategy that has proven successful in involving ‘hard-to-reach’ populations [12].

The relatively high proportion of participants who failed to register interest in the study (see Fig. 1) can be explained by our protocol, which prescribed that contact between the research team and eligible participants had to be initiated by eligible participants: despite expressing interest in the study when contacted by the gatekeepers, many eligible participants did not contact the researchers. Indeed, the recruitment protocol of this study may have been too complicated, thus hindering participation: in particular, parents firstly agreed to receive an information package by the gatekeeper; they then had to read the information, and successively phone the research team on at least one occasion to register their interest and ask for more information. All these steps may have discouraged parents, particularly those less familiar with research and more preoccupied by other priorities of daily living. Practitioners who contributed to the Study Steering Group highlighted that the procedure ‘prevented families from backgrounds with lower levels of education and literacy to make contact with the research team’ (Minutes 6^th^ Meeting, Study Steering Group, Aug 16 2019). Based on the findings from this study and our experience from other studies, we advocate a different strategy that would involve a contact from the research group, provided the family consent to receive information about the study.

However, even within families that registered their interest in the study, only 41% eventually agreed to take part in the study. The steering committee discussed this issue (e.g. Minutes of Steering Group meeting Sep 14 2018) whereby some obstacles to recruitment were identified in the following: (a) a study schedule that may have appeared too strict and demanding for parents; (b) the use of specialised and technical language in describing the study to parents. A series of action points were agreed that included the following: emphasising flexibility around the study schedule (e.g. possibility to skip a weekly session if parents were on holiday); increasing researchers’ flexibility by allowing evening and weekend appointments; changes in the script used by researchers to describe the study to interested parents, and particularly reduced use of technical terms, as well as emphasis on the novelty of the study.

We did not promote this study through media or marketing campaigns, and we made very limited use of social media: The study was only promoted through the social media of the charity involved. However, media and social-media promotion and public information campaigns may be useful, particularly in addressing barriers relating to lack of familiarity with research and low health literacy [12, 68]. Further improvements to the recruitment strategy may involve communication with families. In developing social-media promotion with the charity, we discussed the opportunity to use terminology that promotes trusting relationships (e.g. substituting the term ‘study’ with less intimidating ones, see [26]).

We attempted to incentivise participants by reimbursing parents for travel expenses when they attended lab visits and giving gift vouchers at the end of the study. The latter incentive was meant to cover potential costs of child care (e.g. in looking after participants’ siblings). However, none of the parents who took part in the study described this as an incentive, while some mentioned difficulties in arranging child care as one of the difficulties in taking part. Alternative strategies to support child-care arrangements and its costs may be important in promoting participation. A final consideration concerns the effectiveness of appealing to participants’ altruism: many participants mentioned their desire to contribute to research that may benefit families and infants’ like their own in the future, a finding that is consistent with other research with hard-to-reach populations [12].

In conclusion, the results suggest the feasibility of recruiting this population, but indicate the need to review the recruitment process by improving opportunities for contact and building trusting relationships with the research team, including reduced use of specialist and complex language, and information campaigns using different media. Further ways to minimise practical obstacles (e.g. child-care arrangements) would also be important. In reviewing the recruitment strategy, it will be pivotal to involve and incentivise co-production with third-sector bodies and parents from the communities.

### Retention

Although we recruited a small sample, retention appeared satisfactory: only two infants who had been randomised (17%) did not complete the study. The results however indicate some difficulties in testing infants within the narrow 1-month window at 12 months: two infants started the study when they were past age 13 months (see Fig. 2), due to family engagements that prevented attendance of testing sessions at an earlier age.

Parents who completed the study mentioned challenges that involved the difficulty of making time to attend the study sessions: Some of these challenges concerned ensuring infants attended the sessions in their best condition, i.e. well rested and fed. Other challenges concerned obstacles related to daily life demands, particularly work commitments and arrangement of child care.

Some of these obstacles to retention had been mitigated by delivering the training/control games in the participants’ home: after this option became available, three out of four families recruited in the study opted for delivery of the procedure at home. Only one family declined this option, citing they lived closely to the charity premises where the study was delivered. Feedback from parents emphasised the convenience of avoiding travelling to a lab, having infant’s food and resting facilities at hand, and conducting the study in an environment familiar to the infant. Comparisons between home and lab session are undermined by the fact that only three participants had the programme delivered at home, but so far, these suggest that most of the sessions at home were conducted in adequate conditions without significant distractions and interferences, and the infants were mostly seen in an alert and calm state. Feedback from parents also emphasised the importance of the researchers being flexible and responsive in scheduling study sessions, a factor that other studies indicate facilitate retention of hard-to-reach populations [12]. Feedback from parents indicated that potential facilitators of retention may involve providing more precise information about duration of tasks in advance, breaks that involve infants’ physical activity, as well as refreshments, and provision of a safe testing environment whereby risks for infections are minimised, an aspect that may be particularly salient following the COVID-19 pandemic.

The intervention required relatively long sessions sitting in front of a screen for young infants and all parents that provided feedback reported their child being tired at the end of these sessions. However, parents also reported their child enjoyed the programme. This programme had been developed to be child-friendly, but it had never been trialled with VP infants before: Our results indicate that while the intervention purposely challenges VP infants’ abilities, parents appreciate their VP children’s engagement with the programme, and some parents also reported their gratification in noticing changes in their child’s performance over time.

To summarise, the results indicate that it is possible to retain families of VP infants using the current design. Key facilitators are flexibility and responsiveness to changing situation by the research team, and the possibility to have the training programme delivered in the family’s house. However, implementing this intervention in the future may benefit from increasing the duration of training time: it will thus be pivotal to be able to mitigate obstacles relating to daily life aspects (e.g. parental work commitments).

### Do infants engage with the training or control games?

One of the key questions in testing the feasibility of the ACT with VP infants was whether these infants would engage in the programme adequately. The results of our study indicate a positive answer to this question.

Firstly, infants in both the intervention and control group engaged in the games presented for an adequate number of visits and amount of time. All infants across the two groups completed at least three training or control visits, as we intended. On average, infants in the intervention group accumulated approximately 84 min of training, while infants in the control group watched the non-interactive displays for 75 min in total, on average. The training time in this study was remarkably similar to the training time accumulated by typically developing infants in previous studies that prescribed the same study schedule as our study [6, 74]. Notably, every infant in the training group had accumulated at least 66 min of training time. Furthermore, across the study, all infants in the intervention group completed at least three games from each of the categories we had devised (search for a target among distracters, short-term memory, goal maintenance), demonstrating they had received an adequate amount of training across these domains of attention control (see also Table 4). A consideration for future studies is the short time and small number of infants that completed the training or control games after the pre-test assessment (see Table 2): since the results suggest completing this training/control session was challenging, it may be reasonable to only deliver it when infants have had the time to rest.

Another key index of engagement was trained infants’ performance during training. The results (see Fig. 4) indicated that trained infants generally improved their performance across training visits, indicating that they were progressing and increasingly mastering these tasks.

In conclusion, the results indicate VP infants in this study engaged in the ACT games: The training time accumulated by VP infants was consistent with that reported in other studies involving typically developing infants of the same age [6, 73]; VP infants increased their performance during training, an indication they were adequately engaging with the training material.

### Acceptability of study processes: randomisation, baseline and outcome assessments

Another aim of this study was to test processes we intend to use in a larger trial. The results indicated that randomisation was acceptable: the rationale for randomisation was explained before parents agreed their child to take part, and feedback received from parents indicated they appreciated this rationale, as well as the fact that their child’s allocation was not disclosed at the end of their participation (see Table 5).

This study also investigated how feasible and acceptable was the battery of pre- and post-test we had devised. This battery included several measures of attention and general development, and its delivery lasted over an hour on average. Despite the length and the nature of the testing battery, whereby tasks challenged infants’ socio-cognitive skills, all the VP infants that attended the pre- and post-sessions completed most of the tasks. Indeed, only one infant in the intervention group did not complete two tasks (both tasks from the ESCS). Feedback from parents did not indicate the length and the nature of these assessments to be problematic.

### Quality of eye-tracking measures

Eye-tracking is a research technology that affords several advantages when applied to the study of gaze behaviour, such as high temporal and spatial resolution, and the automated processing of large amount of data that can be readily used in analyses. This technology has also been adopted in the study of infants’ behaviour and cognition, but some methodological challenges remain and have been relatively less investigated [77].

The recording of a participant’s gaze direction relies on correct detection of a series of variable (e.g. the pupil position), which can be disrupted by infants being fidgety and abruptly changing their head and eye position. In assessing the quality of data collected among our sample of VP infants, we focused on two indicators: precision and robustness. Our results suggested moderate quality of eye-tracking data among VP infants.

However, this issue is unlikely to have significantly interfered with the training and its effectiveness, since training tasks are not sensitive to data quality [6]. Furthermore, results reported in previous sections indicate that VP infants’ training times were very similar to those of typically developing infants, and indices of performance also followed consistent patterns that indicated infants’ improvement, hence engagement with the tasks. Were the training tasks significantly affected by lower quality of eye-tracking recordings, we would have expected infants’ engagement to follow more haphazard patterns.

Nonetheless, the results of moderate quality of eye-tracking data suggest that future studies comparing performance of VP and typically developing infants in eye-tracking tasks (e.g. habituation tasks) need to monitor and report the quality of the data collected. Lower quality of data collected among VP infants may bias the results, inflating group differences when VP infants are compared to typically developing infants in eye-tracking task outcomes [77]. Studies that plan to compare performance of VP infants with other groups might have to mitigate factors that can reduce quality of data such as infants’ fidgetiness, for example by ensuring infants are tested when in a calm and alert state.

### Limitations of the study

Our results are limited by the small number of participants, and their homogeneity: most of the VP infants in the study came from higher socio-economic status households, at least according to the educational attainment of the main caregiver. Parents and caregivers from different backgrounds may face more and diverse challenges in taking part in a similar intervention. It will also be important to further investigate the engagement with the ACT intervention of VP infants from different backgrounds, who may differ in temperament and reactivity from an early age [18].

## Conclusions

This is, to the best of our knowledge, the first study that attempts to deliver a cognitive training programme to VP infants. Our results suggest the feasibility of delivering the ACT intervention to VP infants when they are approximately 1 year of age from their due date. However, the results also indicate challenges in recruiting sizeable and diverse samples, which will have to be addressed in future studies. To mitigate these challenges, the study indicated some processes that were successful in recruitment (e.g. trusting relationships with gatekeepers; appeal to participants’ altruism) and in retention (e.g. flexibility in scheduling visits; delivery of the training at home). The results indicate that VP infants engaged successfully with the training, accumulating adequate total training times for their age and improving performance across training visits.

## Supplementary Information

**Additional file 1: S1**. Description of training tasks and performance indicators. **S2**. Multilevel growth models of performance Z scores. **S3**. Results of multilevel growth models on trained infants (*n* = 5). **S3.1**. Z scores in the Goal Maintenance task. **Table S3.1**. Model parameters of the random intercept model of Z performance scores in the goal maintenance task. **S3.2**. Z scores in the Target Search tasks. **Table S3.2**. Model parameters of the random slope model of Z performance scores in the target search tasks. **S3.3**. Z scores in the Short Term Memory tasks. **Table S3.3**. Model parameters of the random slope model of Z performance scores in the short term memory tasks

## Data Availability

The study involved a small number of participants from a defined geographical area and with specific clinical characteristics. Therefore, there are some risks that participants may become identifiable by linking information about them, and therefore data have not been publicly deposited. However, the first author will consider requests for anonymised selected data.

## Abbreviations

ACT: Attention Cognitive Training
ADHD: Attention deficit with hyperactivity disorder
EFs: Executive functions
ESCS: Early Social Communication Scales
IBQ: Infant Behavior Questionnaire
NICU: Neonatal intensive care unit
SES: Social economic status
VP: Very preterm
