# Correction to: Very preterm infants engage in an intervention to train their control of attention: results from the feasibility study of the attention control training (ACT) randomised trial

**DOI:** 10.1186/s40814-021-00943-8

**Published:** 2021-11-11

**Authors:** Oliver Perra, Sam Wass, Alison McNulty, David Sweet, Kostas A. Papageorgiou, Matthew Johnston, Delfina Bilello, Aaron Patterson, Fiona Alderdice

**Affiliations:** 1 School of Nursing and Midwifery, Queen’s University Belfast, Medical Biology Building, 97 Lisburn Road, Belfast, BT9 7BL UK; 2 Centre for Evidence and Social Innovation, Queen’s University Belfast, Medical Biology Centre, 97 Lisburn Road, Belfast, BT9 7BL Northern Ireland, UK; 3 School of Psychology, University of East London, London, UK; 4 TinyLife, The Premature Baby Charity for Northern Ireland, Belfast, Northern Ireland, UK; 5 Health and Social Care Belfast Trust, Belfast, Northern Ireland, UK; 6 School of Psychology, Queen’s University Belfast, Belfast, UK; 7 Nuffield Department of Population Health, University of Oxford, Oxford, UK

**Correction to: Pilot Feasibility Stud 7, 66 (2021)**

**https://doi.org/10.1186/s40814-021-00809-z**

Following publication of the original article [1], the authors reported an omission in the list of authors, having failed to recognise Aaron Patterson for his contribution to data collection.

The original article [1] has been updated.
