# Supplementary material for: Very preterm infants engage in an intervention to train their control of attention: results from the feasibility study of the Attention Control Training (ACT) randomised trial
Source: Pilot Feasibility Stud. 2021 Mar 12;7:66. doi: 10.1186/s40814-021-00809-z (PMC7952829; doi:10.1186/s40814-021-00809-z)
Supplement: Supplementary file 1 — Additional file 1: S1. Description of training tasks and performance indicators. S2. Multilevel growth models of performance Z scores. S3. Results of multilevel growth models on trained infants (n = 5). S3.1. Z scores in the Goal Maintenance task. Table S3.1. Model parameters of the random intercept model of Z performance scores in the goal maintenance task. S3.2. Z scores in the Target Search tasks. Table S3.2. Model parameters of the random slope model of Z performance scores in the target search tasks. S3.3. Z scores in the Short Term Memory tasks. Table S3.3. Model parameters of the random slope model of Z performance scores in the short term memory tasks [file 40814_2021_809_MOESM1_ESM.docx]

## Very preterm infants engage in an intervention to train their control of attention: results from the feasibility study of the Attention Control Training (ACT) randomised trial

## Supplementary Material

### S1. Description of training tasks and performance indicators

| **Goal Maintenance 1: Fly me.**  **Task description**: A pink shape appeared on the screen: when the child fixated it, face-like features appeared on the shape (eyes and mouth) and it moved across the screen. Distractors appeared moving in different directions. When the child looked away from the target character, it stopped and its face-like features faded out while at the same time the distractors faded out from the screen. The target character resumed moving and its face-like features reappeared when the child fixated it again. The distractors changed adaptively.  **Performance indicator**: We calculated the proportion of infants’ fixation to the target weighted by the difficulty level: since more difficult levels presented more distracters, infants’ fixation to the target was given more weight when displayed while presented with more challenges. | 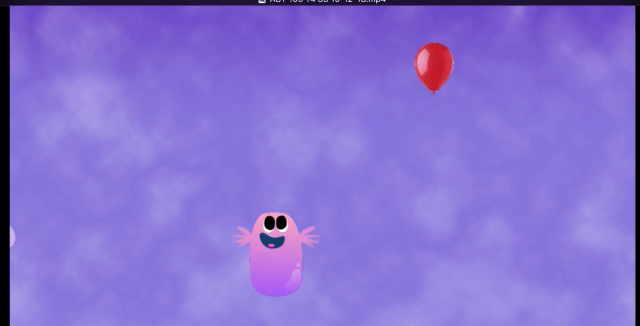 |
| --- | --- |
| **Goal Maintenance 2: Butterfly**.  **Task description**: A target (a butterfly) was presented on the screen. When the child fixated the butterfly, the butterfly flied across the screen, while distractors (a house, a tree, clouds) scrolled in the opposite direction. When the child looked to any of the distractors, they disappeared and only the butterfly, now static, remained on screen. On re-fixating the butterfly, it re-commenced moving and the distractors re-appeared and continued scrolling. The salience of the distractors changed adaptively, including faster, larger and more densely packed objects.  **Performance indicator**: Proportion of infants’ fixation times. However, only two infants completed this task across the training, and only one of them completed it in two visits. Since information provided by this task was limited, we did not consider the results in further analyses. |  |
| **Short Term Memory 1: Puzzle memory**.  **Task description**: A character moved into one out of two (or more) squares, then disappeared behind a screen. The screen then became black and a central stimulus appeared (a ball moving from top midline to bottom) in order to attract infants’ attention. After this, the two squares reappeared covered by the screen. After the infant looked at the square where this character had been, the character was revealed and stayed inside the square. A new character appeared, moved into the other square, the screen became black and the central stimulus (moving ball) was displayed. After this, the two squares reappeared: the first character was still visible inside the square, while the other square was covered by the screen. When the infant looked at the square where the novel character was, the character popped out producing a sound.  **Performance indicator**: We calculated the reciprocal of the response time taken to look at the correct target. Thus, infants who were faster in meeting task demands obtained higher scores. | 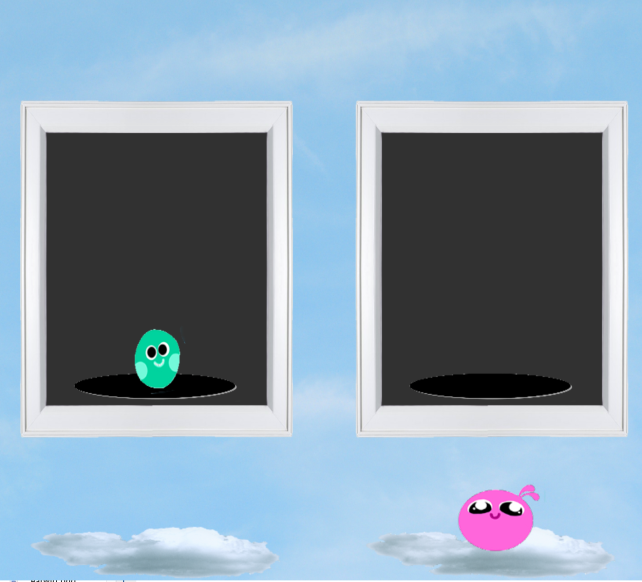 |
| **Short Term Memory 2: Windows**.  **Task Description:** When the infant fixated the target (an animal in a window), an animation showed the target disappearing into one of several windows, which were then covered with curtains. A fixation target (a flower) appeared elsewhere on the screen and rotated when the infant looked at it. After a delay period, the fixation target disappeared. If the infant looked back to the window behind which the target had disappeared, she received an animation as a reward. The number of windows, the salience of the distractors, and the length of the delay changed adaptively.  **Performance indicator**: We calculated the inverse of the response time taken to look at the correct target. Thus, infants who were faster in meeting task demands obtained higher scores. |  |
| **Short Term Memory 3: Tausendfuss**  **Task Description:** An animated character appeared inside a square, moved and made a sound, then disappeared behind a screen that covered both squares. A central stimulus than appeared in the top midline of the screen (a moving spiral). If the infant looked back at the square where the animated character had appeared, the character popped out and made a noise. Following this, when the infant looked at the other empty square, a new animated character appeared. This was also covered by a screen, while the first character remained visible in the other square. A central stimulus appeared again. After this, the second novel character popped out of the covered square if the infant looked at it for a pre-defined amount of time.  **Performance indicator:** We calculated the reciprocal of the response time when infants identified the correct target weighted by the difficulty level. | 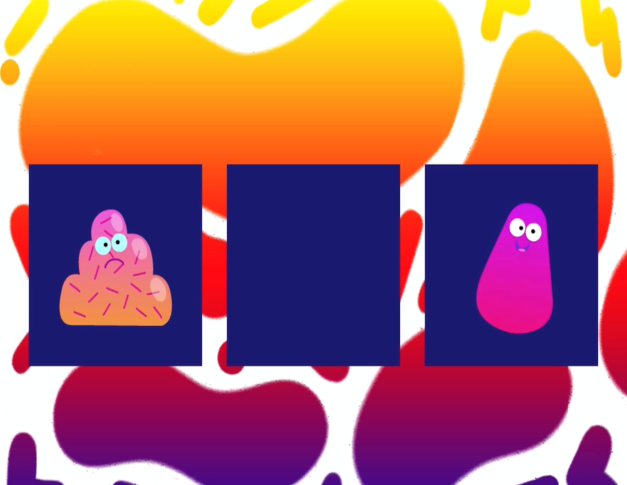 |
| **Short Term Memory 4: Three Little Maids.**  **Task description:** A character appeared on the screen, which was successively covered by one of two (or more) pots that moved into the screen. After this, a central fixation target appeared on the screen to attract the infants’ attention to midline (a moving cartoon spider). After the central fixation stimulus disappeared, the child was rewarded by an animation if she looked back (within a pre-set time limit of 10 s) to the pot in the location where the original character had been. The task changed adaptively by increasing the number of pots on the screen from which to select the target.  **Performance indicator:** We calculated the inverse of the time it took a child to look back at the correct target. | 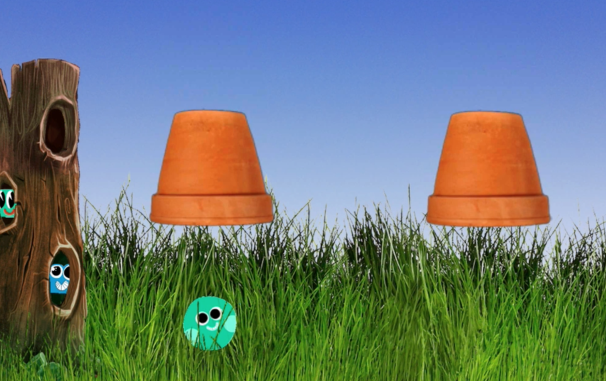 |
| **Target Search 1: Stars**.  **Task description:** One of five possible targets (cartoon characters in brightly coloured stars) were presented on screen together with eight distractors (smaller stars, planets, clouds). If the infant looked to the target within 3000 ms, she received an animation as a reward. The target changed from trial to trial. The salience of the distractors changed adaptively. At lower difficulty levels, the eight distractors were smaller, static, and identical to each other and dissimilar from the targets. At higher difficulty levels, they were more varied, moving, brightly coloured, and similar to the targets.  **Performance indicator:** We calculated the reciprocal of the response time infants employed in finding the correct target weighted by the difficulty level. In this way, infants obtained higher scores for succeeding in the task faster when the task presented higher demands. |  |
| **Target Search 2: Suspects**.  **Task description**: One of two possible targets (either an elephant or a chicken) was presented with one or more distractor items of the same size. When the infant looked at the target within a time limit, she received an animation as a reward. The same target was then re-presented with other distractor(s). The number of distractors varied adaptively with performance; at higher performance levels, more distractors were presented. Between blocks of 12 trials, the target changed: where previously the infant had received a reward for looking to the elephant, she was successively rewarded for looking to the chicken. At higher difficulty levels, the target from the previous block was presented concurrently with the target from the current block (a conflict trial); at lower difficulty levels, only novel distractors were presented (non-conflict).  **Performance indicator:** We considered number of windows displayed in the search task, which increased adaptively during the task. Thus, this measure indicated that infants had successfully mastered the task at lower levels of difficulty and progressed to more challenging displays. |  |
| **Target Search 3: Disengagement.**  **Task description**: A central moving stimulus appeared on the screen (a colourful flower), and after that, two characters appeared. If the infant watched at one of the two chosen at random by the computer script, a rewarding animation was displayed. In successive trials, the infant received the animation every time she looked at the pre-set target character. The number of distractors increased adaptively.  **Performance indicator:** We considered the difficulty level of stimuli presentations: these changed adaptively: thus, a higher difficulty level indicated the infant had successfully mastered the task at lower levels. | 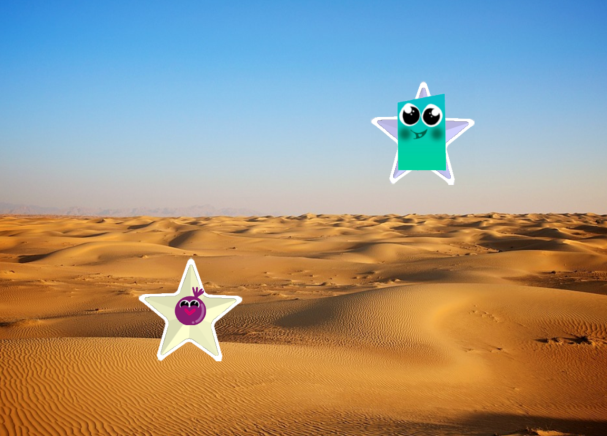 |

### S2. Multilevel growth models of performance Z scores.

Multilevel growth models were used to investigate changes in performance Z scores collated across different types of training tasks in each visit.

The analyses considered that Z scores at each visit *j* were nested within each individual *i.* The order of visits was centred at the training visit scheduled at the end of the pre-test assessment. We started the analyses with **a random intercept model**. This model estimated the average Z scores at the start of the study and the rate of change of these scores across training visits of the n=5 participants, assuming that while participants varied in their initial scores, they displayed a similar rate of change across visits. This model was compared with a **random slope model,** which assumes that participants displayed different rates of change in Z scores across training occasions. These models are nested, so they can be formally compared using *Likelihood Ratio* tests. We retained the random slope model if the Likelihood Ratio test indicated significant improvement of model fit when including the additional parameter of this model.

The **random intercept model** is formally expressed by equation:

Z_ij_= β_0_ + β_1_t_j_ + u_0i_ + e_ij_

whereby:

Z_ij_ represents the performance Z score of participant *i* during visit *j* ;

β_0_ represents the overall initial status (i.e. *intercept*) of Z scores averaged across individuals, or else the expected Z score at visit 0 (the visit at the end of the baseline assessment);

β_1_ represents the rate of change (i.e. *slope*), or growth rate of Z scores from one weekly visit to another. In a random intercept model the assumption is that all individuals display a similar rate of change.

u_0i_ represents an individual-level random effect which results from unmeasured individual characteristics. This is assumed to approximate a normal distribution with mean = 0 and variance $\sigma_{u0}^{2}$, with the latter representing between-individual variance in the Z scores, after accounting for linear effects associated with training visits.

e_ij_ represents an occasion-specific residual resulting from unmeasured time-varying effects. The distribution of this parameter is assumed to approximate a normal distribution with mean = 0 and variance $\sigma_{e}^{2}$, with the latter representing intra-individual variation in the Z scores.

The **random slope model** is formally expressed as:

Z_ij_= β_0_ + β_1_t_j_ + u_0i_ + u_1i_ t_j_ + e_ij_

Compared with the previous random intercept model, the random slope model contains an additional parameter u_1i_ which interacts with time. This parameter represents inter-individual variation in the growth rate across visits. The two individual-level random effects in this model, u_0i_ and u_1i_ are assumed to follow a bivariate normal distribution with mean=0 and variances $\sigma_{u0}^{2}$ and $\sigma_{u1}^{2}$, with the latter representing residual variance around the Z scores rate of change. A further parameter that can be modelled in the bivariate normal distribution of u_0i_ and u_1i_ is σ_u01_, which represents a covariance between individuals’ initial Z scores and their rate of change. We initially tested a model that did not include the covariance term, and compared this with a model including this term using likelihood ratio tests.

**S3: Results of multilevel growth models on trained infants (n = 5)**

**S3.1: Z scores in the Goal Maintenance task**

The comparison of the random intercept model with the random slope model did not indicate significant improvement of model fit Likelihood Ratio *χ^2^(1)* = 0.89, *p* = .34. We thus retained the random intercept model as the final (best fitting) model. Model parameters are reported in Table S3.1.

*Table S3.1: Model parameters of the random intercept model of Z performance scores in the goal maintenance task.*

| Fixed effects | | | | | | |
| --- | --- | --- | --- | --- | --- | --- |
|  |  | *Coef* | *95%* | *CI* | *z* |  |
| Initial Status | β_0_ | **-0.95** | **-1.55** | **-0.35** | **-3.11** |  |
| Rate of Change | β_1_ | **0.41** | **0.23** | **0.59** | **4.50** |  |
| Variance Components | | | | | | |
|  |  | *Coef.* | *95%* | *CI* |  |  |
| Within-person | $\sigma_{e}^{2}$ | 0.61 | 0.45 | 0.84 |  |  |
| Between-Person: Initial Status | $\sigma_{u0}^{2}$ | 0.27 | 0.07 | 1.13 |  |  |

Significant parameters (at *p* < .05) are in bold.

Number of observed scores: 82; Average observations per participant: 16.4 (range 8 – 30)

Model’s Wald *χ^2^(1)* = 20.26; *p* < .001

**S3.2: Z scores in the Target Search tasks**

The random slope model provided improved model fit compared to the random intercept model, Likelihood Ratio *χ^2^(1)* = 54.12, *p* < .001. We thus accepted the former model as the best-fitting model. Model parameters are reported in Table S3.2

*Table S3.2: Model parameters of the random slope model of Z performance scores in the target search tasks.*

| Fixed effects | | | | | | |
| --- | --- | --- | --- | --- | --- | --- |
|  |  | *Coef* | *95%* | *CI* | *z* |  |
| Initial Status | β_0_ | **-0.73** | **-1.08** | **-0.38** | **-4.10** |  |
| Rate of Change | β_1_ | **0.40** | **0.04** | **0.76** | **2.20** |  |
| Variance Components | | | | | | |
|  |  | *Coef.* | *95%* | *CI* |  |  |
| Within-person | $\sigma_{e}^{2}$ | 0.43 | 0.36 | 0.51 |  |  |
| Between-person: Initial Status | $\sigma_{u0}^{2}$ | 0.11 | 0.02 | 0.67 |  |  |
| Between-Person: Rate of Change | $\sigma_{u1}^{2}$ | 0.16 | 0.04 | 0.60 |  |  |

Significant parameters (at *p* < .05) are in bold.

Number of observed scores: 265; Average observations per participant: 53 (range 40 – 66)

Model’s Wald *χ^2^(1)* = 4.85; *p* = .028

The comparison of the random intercept model without a covariance and the model with a covariance did not indicate significant improvement of model fit Likelihood Ratio *χ^2^(1)* = 2.99, *p* = .08. We thus retained the random intercept model without a covariance between the two between-person variances as the best fitting model.

**S3.3: Z scores in the Short Term Memory tasks**

The comparison of the random intercept model with the random slope model did not indicate significant improvement of model fit, Likelihood Ratio *χ^2^(1)* = 0.46, *p* = .50. We thus retained the random intercept model as the final (best fitting) model. Model parameters are reported in Table S3.3.

*Table S3.3: Model parameters of the random slope model of Z performance scores in the short term memory tasks.*

| Fixed effects | | | | | | |
| --- | --- | --- | --- | --- | --- | --- |
|  |  | *Coef* | *95%* | *CI* | *z* |  |
| Initial Status | β_0_ | -0.27 | -0.62 | 0.08 | -1.52 |  |
| Rate of Change | β_1_ | 0.12 | -0.01 | 0.25 | 1.79 |  |
| Variance Components | | | | | | |
|  |  | *Coef.* | *95%* | *CI* |  |  |
| Within-person | $\sigma_{e}^{2}$ | 0.94 | 0.79 | 1.10 |  |  |
| Between-person: Initial Status | $\sigma_{u0}^{2}$ | 0.04 | 0.01 | 0.22 |  |  |

Number of observed scores: 286; Average observations per participant: 57.2 (range 32 – 72)

Model’s Wald *χ^2^(1)* = 3.19; *p* = .074
